# A descriptive, retrospective case series of patients with factitious disorder imposed on self

**DOI:** 10.1186/s12888-021-03582-8

**Published:** 2021-11-23

**Authors:** Antoine Bérar, Guillaume Bouzillé, Patrick Jego, Jean-Sébastien Allain

**Affiliations:** 1 Univ Rennes 1, CHU Rennes, Service de Médecine Interne et Immunologie Clinique, 2 rue Henri Le Guilloux, 35 033 Rennes, France; 2 Univ Rennes, CHU Rennes, INSERM, LTSI – UMR 1099, Rennes, France; 3 Univ Rennes 1, CHU Rennes, Service de Médecine Interne et Immunologie Clinique, Inserm, IRSET (Institut de recherche en santé, environnement et travail) - UMR_S 1085, F-35000 Rennes, France; 4 CHU Rennes, Service de Médecine Interne et Immunologie Clinique, Inserm, CIC 1414, F-35000 Rennes, France; 5 CH Saint Malo, F-35400 Saint Malo, France

**Keywords:** Deception, Factitious disorder imposed on self, Malingering, Medically unexplained symptoms, Munchausen syndrome, Somatic symptom disorder

## Abstract

**Background:**

Despite cases of factitious disorder imposed on self being documented in the literature for decades, it appears to remain an under-identified and under-diagnosed problem. The present study aimed to explore factitious disorder imposed on self in a series of French patients.

**Methods:**

Patients 18 years old and over with factitious disorder imposed on self were retrospectively included by two independent reviewers according to DSM-5 criteria in Rennes University Hospital for the period 1995 to 2019. Patients were identified from a clinical data warehouse.

**Results:**

49 patients with factitious disorder imposed on self were included. Among them, 36 (73.5%) were female. The average age at diagnosis was 38.4 years. The 16 patients with a health-related profession were all female. Direct evidence of falsification was found in 20.4% of cases. Falsification was mainly diagnosed on the basis of indirect arguments: history of factitious disorder diagnosed in another hospital (12.2%), extensive use of healthcare services (22.4%), investigations that were normal or inconclusive (69.4%), inconsistent or incomplete anamnesis and/or patient refusal to allow access to outside information sources (20.4%), atypical presentation (59.2%), evocative patient behaviour or comments (32.7%), and/or treatment failure (28.6%). Dermatology and neurology were the most frequently involved specialities (24.5%). Nine patients were hospitalized in intensive care. Some of them received invasive treatments, such as intubations, because of problems that were only reported or feigned. The diagnosis of factitious disorder imposed on self was discussed with the patient in 28 cases (57.1%). None of them admitted to making up the disorder intentionally. Two suicide attempts occurred within 3 months after the discussion of the diagnosis. No deaths were recorded. 44.9% of the patients returned to the same hospital at least once in relation to factitious disorder imposed on self.

**Conclusions:**

The present study reinforces data in favour of a predominance of females among patients with factitious disorder imposed on self. This diagnosis is difficult and is based on a range of arguments. While induced cases can be of low severity, cases that are only feigned can lead to extreme medical interventions, such as intubation.

**Supplementary Information:**

The online version contains supplementary material available at 10.1186/s12888-021-03582-8.

## Background

Factitious disorder imposed on self (FDIS) is defined by the fifth edition of the *Diagnostic and statistical manual of mental disorders* (DSM) as “falsification of physical or psychological signs or symptoms, or induction of injury or disease, associated with identified deception” [1]. Individuals pretend to be ill and hide the artificial origin of their disorder. According to both the DSM-5 and the eleventh revision of the *International Classification of Diseases*, in FDIS the deceptive behaviour is not primarily driven by external rewards [1, 2]. In contrast, in the case of malingering, obvious external rewards or incentives motivate the behaviour [2]. The rewards experienced in FDIS are complex and far less intelligible. One of the primary incentives could be to appease a need for attention by acting and being treated like a patient. There could also be some enjoyment in challenging and misleading doctors in finding a diagnosis or an effective treatment [3]. FDIS should also be distinguished from somatic symptom disorder [4].

FDIS can be encountered in all medical specialities [5]. Self-induced skin lesions, hypoglycaemia from hidden insulin injections or chest pain have often been reported [5]. Anaemia due to iron deficiency secondary to self-inflicted bleeding is a classic form of FDIS known in France as Lasthénie de Ferjol syndrome [6]. Munchausen syndrome is a form of FDIS characterized by its severity and frequent surgical presentations [7]. However, FDIS remains an under-identified and under-diagnosed problem. Most of the research assessing FDIS is based on literature reviews or long-standing systematic samples [5, 8–19], which suggest a predominance of females and a marked prevalence of health-related professions in the population with FDIS. Several isolated cases reports describe serious events or even deaths, but there is no data available on the prevalence of these complications [20]. Likewise, optimal management is not codified. One of the largest systematic samples of FDIS patients dates back almost 20 years [21]. Its authors called for further research, particularly to confirm the female predominance in FDIS, which could not be reliably concluded from case reports. FDIS was subsequently studied in a recent systematic sample [22]. Nevertheless, more than half of the subjects included in this research had an external motivation, in this case the prospect of financial gain, which could be an exclusion criterion according to the DSM-5 definition if it were the primary motivating factor. In fact, better description of FDIS requires more systematic samples. The primary objective of the present work was to study the clinical characteristics of FDIS. The secondary objectives were to study the characteristics of patients with FDIS, the diagnostic methods, any serious complications, and the management of the disorder and the consequences resulting from this management (especially the risk of suicide attempt after the diagnosis).

## Methods

### Study design and patient selection

Approval was received from the Rennes University Hospital ethics committee (Comité d’éthique du CHU de Rennes, number 19.62, 2019, September 23rd). The need for informed consent was waived by the ethics committee, due to the retrospective nature of the study and the particular nature of the disorder. Only non-opposition to participating was to be collected from the patients, so as not to risk to break the trust between them and the care teams.

Information on the patients included was derived from the hospital clinical data warehouse, eHOP software, which gathers medical and paramedical documents, including medical reports, produced during stays at Rennes University Hospital, France [23]. These stays were full hospitalizations, outpatient hospitalizations, or consultations. A full-text search was performed on this entire database, from 1995 to 2019. The terms used were “Munchausen”, “factitious disorder”, “pathomimia”, “Ferjol”, and their derivatives (spelling variants and plurals).

The diagnosis was made in accordance with DSM-5 criteria after independent perusal of the medical records by two investigators (AB, JSA). The DSM-5 criteria are (i) falsification of physical or psychological signs or symptoms, or induction of injury or disease, associated with identified deception, (ii) the individual presents himself or herself to others as ill, impaired or injured, (iii) the deceptive behaviour is evident even in the absence of obvious external rewards and (iv) the behaviour is not better explained by another mental disorder, such as delusional disorder or another psychotic disorder [1]. Patients under 18 years of age and patients with factitious disorder imposed on another were excluded from the study.

### Data collection

The parameters analysed were the medical specialities dealing with FDIS cases, modes of falsification (falsely reported, feigned or induced signs or symptoms), types of signs or symptoms (physical or psychological), patient socio-demographic characteristics (age at diagnosis, sex, professional status, profession if applicable, any link of the present or a past profession or studies to the medical environment, marital status, parental status), patient history and comorbidities (psychiatric and non-psychiatric, including the existence of a possible history of undiagnosed FDIS), body mass index (BMI), factors leading to the diagnosis of FDIS and place of the diagnosis, hospitalization in an intensive care unit, management of the disorder once diagnosed (whether or not the diagnosis had been discussed, referral to psychiatric or psychological care), the patient’s reaction if the diagnosis has been discussed and evolution of the disorder once diagnosed (whether or not a break in follow-up or a suicidal gesture occurred following the discussion of the diagnosis) and occurrence of death. In the absence of other sources, information on lifestyle, life history and medical history was reported by the patients themselves. Different expressions of FDIS could be collected from the same patient, who could thus, for example, be referred to different specialities or could have several modes of presentation. In this case, management and follow-up items (e.g. the course of the FDIS or the occurrence of a suicide attempt) were determined from the first diagnosis. It was concluded that there was no psychiatric history only if the patient had undergone an assessment by a psychiatrist. Otherwise, the information was considered to be missing. The existence of a possible history of undiagnosed FDIS was explored by reading all the medical reports that preceded the current diagnosis of FDIS. It was retained when a presentation was suggestive of FDIS but a differential diagnosis was also possible.

### Statistical analysis

Descriptive statistics were computed. Categorical variables were presented as numbers with the corresponding percentages. Quantitative variables were presented as means and standard deviations. Minimum and maximum age values were collected.

## Results

**Fig. 1:**
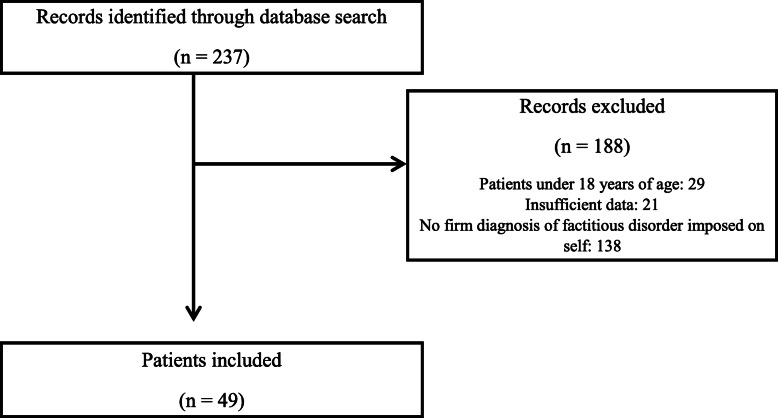
Flow diagram

We identified 237 patients involved at least once for one or more of the predefined keywords (“Munchausen”, “factitious disorder”, “pathomimia”, “Ferjol”, and their derivatives). Among them, 49 (20.7%) were included (Fig. 1).

The general characteristics of the subjects are specified in Table 1. The mean age was 38.4 years (minimum 19 years, maximum 61 years). Among the 34 patients with a known occupation, 16 (47.1%) had a health-related profession or education and all these were female. They included 9 nurses. The other patients with a health-related occupation were an ambulance driver, care assistants (2 people), a life-support worker, a veterinary student, a medical secretary and a hospital administration manager. Sixteen patients (32.7%) were identified with a possible history of undiagnosed FDIS.

**Table 1:** Characteristics of subjects with FDIS

**Sex (*n*** **= 49)**	
Female	36 (73.5%)
Male	13 (26.5%)
**Age at diagnosis (*n*** **= 49)**	
Total population (mean)	38.4 years (SD: 12.6 years)
Female (mean)	37 years (SD: 11.9 years)
Male (mean)	42.5 years (SD: 14.1 years)
**Body mass index (*n*** **= 31)**^a^	
Under 18.5 kg/m^2^	7 (22.6%)
18.5–25 kg/m^2^	10 (32.3%)
25–30 kg/m^2^	6 (19.4%)
Over 30 kg/m^2^	8 (25.8%)
**Parental status (*n*** **= 44)**^a^	
At least one child	22 (50%)
No children	22 (50%)
**Marital status (*n*** **= 37)**^a^	
In a relationship	22 (59.5%)
Single	8 (21.6%)
Divorced, separated	5 (13.5%)
Widower	2 (5.4%)
**Employment status (*n*** **= 43)**^a^	
Working	16 (37.2%)
Unemployed	17 (39.5%)
Student	6 (14%)
Retired	2 (4.7%)
Prolonged work stoppage	2 (4.7%)
**Psychiatric history (*n*** **= 34)**^a b^	
Depression	20 (58.8%)
Bipolar disorder	1 (2.9%)
Chronic psychosis	0 (0%)
Personality disorder	6 (17.6%)
Eating disorders	4 (11.8%)
Suicide attempt	9 (26.5%)
History of psychiatric hospitalization or follow-up, diagnosis unspecified	7 (20.6%)
None	3 (8.8%)
**Addiction (excluding smoking) (*n*** **= 20)**^a^	
Yes	6 (30%)
Stopped	3 (15%)
Active	3 (15%)
No	14 (70%)

^a^Missing data

^b^Several possibilities for each patient

Numerous specialities were called on in FDIS as shown in Table 2. Dermatology and neurology were the most frequently involved specialities (24.5%). Nine of the 12 diagnoses involving dermatology (75%) were made in an outpatient setting. In all cases, there were skin lesions caused by mechanical action on the skin. 6 patients presented Lasthénie de Ferjol syndrome (factitious anaemia).

**Table 2:** Specialities called on in FDIS (*n* = 49)^a^

Dermatology and Plastic Surgery	12 (24.5%)	Skin lesions or wound deterioration induced by mechanical action (*n* = 12)
Neurology	12 (24.5%)	Seizures reported and/or feigned (*n* = 5), gait disorders reported and/or feigned (*n* = 4), unconsciousness feigned (*n* = 3)
Haematology	6 (12.2%)	Anaemia induced (*n* = 6)
Microbiology and Infection	6 (12.2%)	Sepsis induced (*n* = 2), soft tissue infection induced (*n* = 1), fever feigned (*n* = 1), septic arthritis induced (*n* = 1), abscesses induced (*n* = 1)
Ophthalmology	4 (8.2%)	Acute vision loss reported (*n* = 4)
Cardiology	4 (8.2%)	Chest pain reported (*n* = 2), syncopal episode feigned (*n* = 1), chest pain and history of pulmonary embolism reported (*n* = 1)
Gastroenterology and Abdominal Surgery	4 (8.2%)	Acute abdominal pain reported (*n* = 2), acute abdominal pain and diarrhoea reported (*n* = 1), chronic diarrhoea reported (*n* = 1)
Endocrinology	3 (6.1%)	Hypoglycaemia induced (*n* = 2), history of diabetes reported (*n* = 1)
Urology and Gynaecology	3 (6.1%)	Urinary incontinence reported (*n* = 1), urinary retention feigned (*n* = 1), pelvic pain reported (*n* = 1)
Pneumonology	2 (4.1%)	Asthmatic episodes feigned (*n* = 1), acute respiratory distress feigned (*n* = 1)
Oncology	2 (4.1%)	History of Hodgkin’s disease reported (*n* = 1), history of lung cancer reported (*n* = 1)
Traumatology	1 (2%)	Bone fracture feigned (*n* = 1)

^a^Several possibilities for each patient

The characteristics of the factitious disorders imposed on self are described in Table 3. The list of the factors that led to the diagnosis of FDIS is based on the review by Yates and Feldman and available as supplementary material [5]. Nine hospitalizations in an intensive care unit in relation to FDIS were noted. Among them, two appeared to be caused by induced problems, in both cases hemodynamic failure of septic origin. The other cases were respiratory or neurological failures which were feigned and were not directly life-threatening. The diagnosis of FDIS was discussed with the patient in 28 cases (57.1%). None of them admitted to deceptively producing the symptoms. One patient acknowledged his involvement in the production of skin lesions but denied any intention to deceive. Two suicide attempts occurred within the 3 months after the diagnosis, both within a month. One of them was an intentional drug intoxication, the authenticity of which can nevertheless be doubted, since the patient did not exhibit impaired alertness after having reportedly taken twenty oxazepam tablets, possibly suggesting that it could be another manifestation of FDIS for this patient. The other occurred after a marital breakdown. In the first case, the diagnosis had not been discussed with the patient, while in the second case the patient had been confronted with inconsistencies.

**Table 3:** Clinical characteristics of FDIS

**Factors leading to diagnosis of FDIS (*n*** **= 49)**^a^	
History of factitious disorder diagnosed in another hospital	6 (12.2%)
Extensive use of healthcare services	11 (22.4%)
Investigations normal or inconclusive	34 (69.4%)
Inconsistent, incomplete anamnesis and/or patient refusal to allow access to outside information sources	10 (20.4%)
Atypical presentation	29 (59.2%)
Evidence or indication of falsification	10 (20.4%)
Evocative patient behaviour or comments	16 (32.7%)
Treatment failure	14 (28.6%)
**Mode of presentation of FDIS (*n*** **= 49)**^a^	
Falsely reported only	14 (28.6%)
Feigned	16 (32.7%)
Induced	25 (51%)
**Type of sign or symptom (*n*** **= 49)**^a^	
Physical symptom	11 (22.4%)
Physical sign	46 (93.9%)
Psychological symptom or sign	0 (0%)
**Inpatient hospitalization related to FDIS in the institution (*n*** **= 49)**	
Yes	35 (71.4%)
Including intensive care unit	9 (18.4%)
No	14 (28.6%)
**Place of diagnosis of FDIS (*n*** **= 49)**	
Consultation	13 (26.5%)
Emergency department	9 (18.4%)
Hospitalization	27 (55.1%)
**Discussion of the diagnosis with the patient (*n*** **= 49)**	
Confronting with inconsistencies	8 (16.3%)
Questions asked around self-induction	14 (28.6%)
Notification of the absence of organic diagnosis	6 (12.2%)
Offer of psychological or psychiatric care without discussion of the diagnosis	9 (18.4%)
No discussion of the diagnosis or offer of psychological or psychiatric care	12 (24.5%)
**Discontinuation of follow-up after diagnosis (*n*** **= 40)**^b^	
Yes	32 (80%)
By the doctor	26 (65%)
By the patient	6 (15%)
No	8 (20%)
**Evolution of FDIS in the facility after diagnosis (*n*** **= 49)**	
Absence of recurrence in the facility	12 (24.5%)
Chronicisation of the same disorder or recurrence of FDIS in another mode in the facility	22 (44.9%)
Patient never returned to the institution	15 (30.6%)

^a^Several possibilities for each patient

^b^Missing data

The 8 patients with psychiatric care before the diagnosis of FDIS all continued their follow up. Among the other 41 patients, the diagnosis of FDIS was followed by hospitalization in psychiatry in 3 cases (7.3%), including one without consent. A psychiatric consultation was offered for 22 patients (53.7%), and declined by two of them. Among the other 20, the number of patients who actually participated in this consultation is unknown.

Of the 26 cases where the physician did not offer to continue follow-up after diagnosis, a referral to another health professional was offered in only 10 cases (38.4%).

There were no deaths related to FDIS or following the discussion of the diagnosis.

## Discussion

The present work assessed the medical records of a sample of patients affected by FDIS. This European single-centre sample and the one studied by Jimenez are the two largest systematic samples of FDIS in recent years. The study by Jimenez, based in the USA, favoured a psychiatric approach and did not describe markers associated with the severity of the disorder (hospitalizations in intensive care, death) or the method of diagnosis [22]. Krahn’s robust study in 2003 enrolled 93 patients who were included according to the DSM-IV criteria on the basis of medical records and was also based in the USA [21]. With regard to the literature reviews, the reviews by Yates and Caselli included 455 and 514 subjects respectively, but they had a recruitment bias relating to the compilation of case reports [5, 8]. Indeed, published case reports probably select the most unusual cases of FDIS and therefore do not reflect the entirety of this disorder.

The present study showed a predominance of female patients with FDIS (73.5%), in line with observations already made on this population in previously cited research. It is worth noting that this prevalence is in particular similar to that reported in Krahn’s study (72%). These results cast doubt on the previous assumption that the majority of patients are male, as specified in the DSM-IV criteria – this stance was in fact abandoned in DSM-5. The mean age in our study (38.4 years) is consistent with other research in patients 18 years old and over: 34.2 years and 33.5 years on average for the Yates and Caselli literature reviews respectively, and 34 years for Carney’s sample (42 cases) [9, 11]. Reich’s series (41 cases) and Freyberger’s series (70 cases) included patients under 18 years of age and found an average age of 33 and 31.9 years respectively.

The frequency of a health-related occupation was 47.1% for patients with a known occupation in our study, compared to 68.3% for Reich, 67% for the recent work by Jimenez (49 cases), 57% for Yates, 50% for Carney and 44.1% for Krahn. We cannot exclude a bias overestimating all these results, since the diagnosis of FDIS could be favoured when a health-related profession is known. It is possible that the frequency of health-related occupations in patients with FDIS will decrease over time with the development of the Internet, as nowadays anyone can easily search for medical information or download falsified medical records. With regard to other self-reported data, such as parental or marital status, or psychiatric history, it should be noted that these data may be inaccurate because their veracity could not always be verified.

To our knowledge, BMI among subjects with FDIS has never been studied. The prevalence of overweight and obesity combined was 46.7% in the present study, while it was 47.3% in the over 18-year-old French population in 2012 [24], that is to say on the average date of the diagnosis of FDIS in the present study. A BMI under 18.5 was found for 20% of the patients in our study (including 83.3% women), compared to 3.5% in the French population. This could either reflect a higher frequency of FDIS among patients with eating disorders or a bias related to missing data, as extreme weights may be reported more frequently than weights that are considered normal.

Dermatology was the most frequently involved speciality in the present study (24.5%), along with neurology. This is consistent with recent results [25] but contrasts with the lower prevalence of dermatology cases in the review by Yates and Feldman (9.5%). This difference could be explained by a publication bias in the literature excluding the less spectacular cases and causing underestimation by Yates and Feldman, or by better knowledge of this diagnosis among the Rennes hospital dermatologists, leading to more efficient identification of cases related to this speciality in the study population. It also contrasts with Carney’s study, which did not include any case linked to dermatology. The authors nevertheless suggested that this result could be explained by the recruitment of exclusively hospitalized patients, while FDIS with a dermatological presentation was more a matter of ambulatory management. This hypothesis is consistent with the present results, since in the majority of cases, the diagnosis of FDIS was made in an outpatient setting. Interestingly, two cases related to oncology were highlighted in our study. Cancers, and other similar conditions, may seem difficult to falsify, and yet patients can convince doctors they have cancer, especially by using falsified hospital reports [5, 26–28].

One of the main incentives in FDIS is sometimes considered to be concealment of psychological difficulties by taking on the role of a “somatic” patient, which is more socially acceptable [29]. Nevertheless, FDIS is well-documented in psychiatry [8]. The single-centre nature of the present study in a hospital without a psychiatric unit was probably the reason why we did not identify any cases of falsification of a psychiatric disorder. This could also explain the lack of data regarding the subjects’ social and psychiatric status, and it could be the cause of an underestimation of these factors for a certain number of subjects. In particular, the frequency of personality disorders (20%) was probably underestimated in the present study, since non-psychiatric doctors rarely make this diagnosis. In Jimenez’s work, in which all patients were assessed by a psychiatrist, 33% had a personality disorder, which also seems very low with regard to FDIS. These findings underscore the limited utility of a psychiatric consultation interview as the sole source of information to confirm or rule out FDIS or conclude to a personality disorder. It is also likely that FDIS with a psychiatric presentation is under-diagnosed because it is more difficult to identify. The rare diagnoses of psychiatric disorders necessarily involving an external event make them easier to recognize: post-traumatic stress disorder (which can only occur after exposure to a serious event - death, serious injury), bereavement (following the death of a loved one) [30, 31]. Interviews with relatives can point to a diagnosis when they show that the events reported by the patients never actually existed.

The present results confirm that the diagnosis of FDIS is rarely accepted by patients, as none of them admits to trying to deceive others. In Carney’s work, nearly 10% of the patients admitted to being the cause of the observed clinical manifestations [11]. Reich’s work stands out from other studies, with 12 patients out of 33 (36.4%) acknowledging deceitful behaviour [9].

The two cases of severe haemodynamic failure were reminiscent of the reported cases of deaths resulting from self-induced infection among patients with FDIS [9]. In fact, it has been suggested that the severity of FDIS should be based on whether the medical problem is falsely reported, feigned or induced [5]. However, in the present study, a significant proportion of induced cases were self-inflicted skin lesions of low severity. Conversely, feigned presentations could have serious consequences: for example, a patient who feigned acute respiratory failure was intubated several times. Two suicide attempts occurred within 3 months of the diagnosis of FDIS, although neither of them seemed clearly attributable to the discussion of the diagnosis. Other suicide attempts may not have been known in our general care setting: some patients may have been admitted directly to a psychiatric facility, or may have attempted suicide without medical care thereafter. In Reich’s work, no suicidal behaviour was found after subjects were confronted with the diagnosis of FDIS [9]. More research is needed to study suicide risk in FDIS.

Apart from the acute management of suicide risk, the psychiatric management of FDIS must be a long-term process. However, it is poorly codified. No management technique has been shown to be superior to another [4]. Usually, psychotherapy with individuals presenting FDIS includes the steps of acknowledging and gaining better understanding the problem, developing more effective coping skills, increasing empathy towards people negatively impacted by the falsifications (friends, family, professionals), taking responsibility for one’s own recovery, and developing a helpful support system [32].. Agreement of the diagnosis of FDIS should be sought with the patient [29]. Angry or threatening interactions should be avoided [33]. Management of psychiatric comorbidities is essential, and psychotropic medications may be indicated for this purpose [34].

We cannot exclude a risk of having underestimated the number of cases of patients with FDIS. This risk is inherent in the study of this disorder. Clinicians are trained to believe the medical histories provided by their patients and to use that information when making diagnoses and deciding on treatments. They are not trained to disbelieve patients, which makes it very difficult to suspect deception. When they finally suspect FDIS, evidence of the deception is required to make a definitive diagnosis, but it is not easy to demonstrate. Warning signs are helpful but cannot suffice to reach a diagnosis, as they can also apply to patients with chronic illnesses: for example, these chronic patients may also exhibit high health service utilization, normal test results, atypical presentations or treatment failure. Therefore, when deceptive behaviour has not been formally established, it can seem less risky for a physician, at least in written records, to conclude to a symptom of unknown origin rather than to FDIS. Physicians thus spare their patients the potentially stigmatizing diagnosis of FDIS. They also avoid falling into the trap of diagnosing a FDIS whenever a symptom is difficult to explain. In addition, clinicians are not always familiar with the diagnosis of FDIS, even though some classic diagnoses are well known to practitioners, such as factitious anaemia in France since its first description in 1967 [6]. Besides, a history of possible FDIS was found for 32.7% of the patients, which suggests either a strong epidemiological association between FDIS and similar diagnoses, or missed diagnoses of FDIS. Indeed, the distinction between FDIS, somatic symptom disorder and malingering can be tedious and time-consuming [35]. Doctors are not always accustomed to this approach, or may not have sufficient time to spare (especially in consultation). Finally, it may be difficult to differentiate FDIS from the simple exaggeration or amplification of symptoms. The existence of all of these challenging differential diagnoses can either result in an underestimation or an overestimation of the frequency of FDIS. In our opinion, underestimation of its frequency is more likely given the great caution exercised in diagnosing a FDIS, as discussed above. Reluctance to diagnose FDIS despite sometimes strong evidence had also been reported elsewhere [21]. However, failure to diagnose FDIS may lead to the prescription of unnecessary or even dangerous investigations or treatments.

Another bias is the recruitment of our patients in a hospital, even if some of the subjects only came for consultation. This probably influenced the presentations of FDIS observed, such as the specialities involved, and overestimated the frequency of hospitalizations in intensive care. A study of FDIS diagnoses among general practitioners would certainly yield very different results. For the same reason, the design of our study did not allow for a proper assessment of medical nomadism (peregrination), which is commonly attributed to patients with FDIS.

## Conclusion

The present study explored FDIS in a European hospital sample. It is likely that the frequency of FDIS was underestimated, due in part to differential diagnoses. In all events, our results reinforce the data in favour of a predominance of females among patients with FDIS. Most patients did not have a health-related profession. No deaths were identified, although nine hospitalizations in intensive care units were noted and two suicide attempts were reported after the diagnosis of FDIS. Inference on the basis of warning signs of deceptive behaviour was the most frequently-used approach to identifying possible FDIS or diagnosing FDIS, with few instances of direct observation of deceptive behaviour. This type of approach can increase false positive rates and also decrease clinician confidence in their impressions, since many of the warning signs are also present among chronically ill patients. The more tedious and time-consuming gold standard approaches are difficult to use in clinical settings and highlight the need for innovative use of machine learning to flag patients at high risk, as well as other electronic medical record solutions. The prognosis of FDIS needs to be clarified in further studies. Likewise, its optimal management needs to be better known, as many patients returned to the hospital at least once in relation to their FDIS.

## Supplementary Information

**Additional file 1.**

## Data Availability

The datasets used and/or analysed during the current study are available from the corresponding author on reasonable request.

## Abbreviations

BMI: Body mass index
DSM: Diagnostic and statistical manual of mental disorders
FDIS: Factitious disorder imposed on self

## Declarations

All methods were carried out in accordance with relevant guidelines and regulations.
