# Supplementary figures and images for: A descriptive, retrospective case series of patients with factitious disorder imposed on self
Source: BMC Psychiatry. 2021 Nov 23;21:588. doi: 10.1186/s12888-021-03582-8 (PMC8609835; doi:10.1186/s12888-021-03582-8)

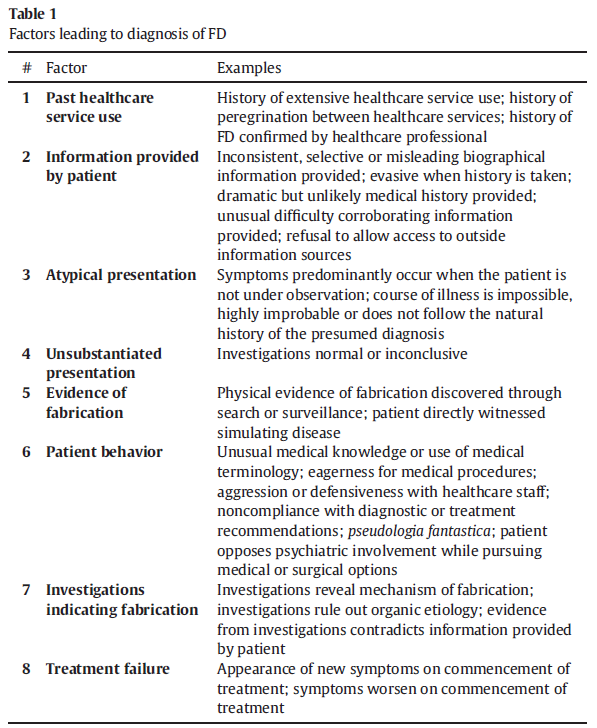

Supplement: Supplementary file 1 — Additional file 1. [file 12888_2021_3582_MOESM1_ESM.png]
